# Efficacy and Safety of a Lidocaine Gel in Patients from 6 Months up to 8 Years with Acute Painful Sites in the Oral Cavity: A Randomized, Placebo-Controlled, Double-Blind, Comparative Study

**DOI:** 10.1155/2015/141767

**Published:** 2015-11-29

**Authors:** Dörte Wolf, Joachim Otto

**Affiliations:** ^1^CardioSec Clinical Research GmbH, Dalbergsweg 21, 99084 Erfurt, Germany; ^2^Chemische Fabrik Kreussler & Co. GmbH, Rheingaustraße 87-93, 65203 Wiesbaden, Germany

## Abstract

Lidocaine is a well-accepted topical anaesthetic, also used in minors to treat painful conditions on mucosal membranes. This randomized, double-blind, placebo-controlled study (registered prospectively as EudraCT number 2011-005336-25) was designed to generate efficacy and safety data for a lidocaine gel (2%) in younger children with painful conditions in the oral cavity. One hundred sixty-one children were included in two subgroups: 4–8 years, average age 6.4 years, treated with verum or placebo and 6 months–<4 years, average age 1.8 years, treated only with verum. Pain reduction was measured from the time prior to administration to 10 or 30 minutes after. In addition, adverse events and local tolerability were evaluated. In group I, pain was reduced significantly after treatment with verum compared to placebo at both time points. In group II, the individual pain rating shift showed statistically significant lower pain after treatment. Only seven out of 161 patients reported an adverse event but none were classified as being related to the study medication. The local tolerability was assessed as very good in over 97% of cases. For painful sites in the oral cavity, a 2% lidocaine gel is a meaningful tool for short-term treatment in the paediatric population.

## 1. Introduction

The use of medicinal products in younger children, even if approved and accepted for decades in adults and older children, often takes place with some uncertainties on the parents' or physician's site. Younger patients not only are smaller and have less weight but also have at least partly different pharmacokinetic characteristics [1]. Although for topically applied and locally acting medicinal products the potential negative consequences of use are considered less important, controlled in-use-data is highly appreciated.

Clinical trials according to accepted rules represent the highest standard of clinical evaluation to prove efficacy and safety but are difficult to perform in younger patients for various reasons [2]. Ethical aspects are more critical and also the design of a study is more demanding, for example, choice of reliable efficacy criteria, especially if laboratory tests or noninvasive measurements are not suitable.

Pain perception shows a high degree of individual variation in intensity, onset, and degree of toleration [3]. In patients not reliably able to specify pain perception, due to their age, it can be very difficult to measure whether a pain medication was successful, especially when the pain is mostly moderate and local.

Lidocaine is a well-established and approved local anaesthetic alone and in combination used in a variety of topically applied preparations, mostly as medicinal product [4]. It is also used to treat painful oral conditions in children, like aphthous ulcers, blisters, teething, or gingivitis.

The objectives of this randomized, double-blind, placebo-controlled, phase IV study were to determine the efficacy and safety of a gel with 2% lidocaine in children from 6 months up to 8 years with painful conditions in the oral cavity as a meaningful tool for short-term treatment in the paediatric population. The verum preparation used is available in Germany for short-term, symptomatic treatment of pain on the oral mucosa, on the gums, or on lips. The dosing recommendation for children is up to 4 times daily as a pea size piece of gel (approximately 0.2 g gel or 4 mg lidocaine hydrochloride).

## 2. Patients and Methods

This randomized, double-blind, placebo-controlled, single-centre, phase IV clinical trial (EudraCT number 2011-005336-25) was initiated after approval by the responsible ethics committee of the Medical Chamber of the German Federal State of Thuringia and by the German National Health Authority (BfArM). The study was conducted in accordance with the World Medical Association Declaration of Helsinki 2013, the Guidelines of ICH Good Clinical Practice (GCP) as well as the requirements of German national drug and data protection laws.

All patients and their parents or legal guardians were given sufficient time and necessary information to consider the benefits and risks of involving the children in the clinical trial before written informed consent was obtained and before assent was sought from the child.

Two different age groups (I: 4–8 years and II: 6 months–<4 years) were included. Children with painful site/s in the oral cavity aged 4 years or older were randomly assigned to be treated either with verum or with placebo, whereas children younger than 4 years were only treated with verum.

### 2.1. Study Medication, Dosage, and Administration

The 2% lidocaine gel (commercially available as Dynexan Mundgel, Kreussler Pharma, Germany) and the placebo preparation (identical composition without lidocaine, both products filled in identical 20 mg tubes to guarantee blindness) were locally applied once by or under supervision of a member of the study staff to the painful site/s as a pea size amount of gel (0.2 g gel) corresponding to 4 mg lidocaine hydrochloride in case of verum.

The whole study took place at a clinical trial facility, specialized in conducting clinical studies with children. Different patient recruitment arrangements, like advertisements in the local newspaper, referrals by paediatricians, and distribution of flyers, were performed to enlarge the number of suitable patients, after obtaining approval by the ethics committee and health authority.

### 2.2. Pain Measurement

The painful sites were defined as open breaks of the oral mucosa, lips, or tongue as a symptom of a variety of mild diseases, or trauma, for example, bites, aphthous ulcer, mouth blisters, cold sores, partial gingivitis, hand-foot-mouth disease, herpes simplex, teething, and trauma from dental braces.

**Figure 1:**
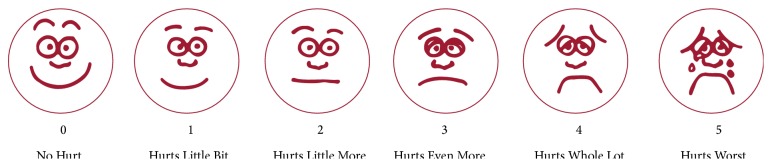
Wong-Baker FACES Pain Rating Scale (reprinted with permission from [9]).

The primary efficacy variable was the pain reduction from T1 (prior to administration) to T2 (10 ± 5 min after application) as assessed by the Wong-Baker FACES Pain Rating Scale (Figure 1). This scale is a self-report measure used to assess the intensity of children's pain. The rating scale was used, because of its suitability for children from 3 years of age on as well as its appropriateness in pre- or nonverbal children. Children were instructed to indicate their pain by pointing to one of the faces. If the child was not old enough and/or not able to use this scale, the assessment of the children's pain by the parents on the same scale was used instead.

A secondary efficacy variable was pain reduction from T1 (prior to administration) to T3 (30 ± 10 min after application, Wong-Baker FACES Pain Rating Scale). This “mixed” analysis of children's and parents' assessments was performed to consider the child's assessment as often as possible according to the protocol and statistical analysis plan (SAP). To investigate whether this approach was reliable, an additional analysis was performed with assessments of the same person (either child, if available from T1 and T2 (or T3), or parent, if the child's assessment was missing at one or two points of time). Additionally, assessment of satisfaction (5-point verbal rating scale with “very unsatisfied,” “somewhat unsatisfied,” “slightly satisfied,” “satisfied,” and “very satisfied”) and assessment of local tolerability by the investigator (“poor,” “moderate,” “good,” and “very good”) were evaluated.

### 2.3. Sample Size Estimation

Sample size calculation was performed within a parametric test scenario. In age group I, a sample number of 64 subjects per treatment arm was considered sufficient to obtain reliable data (assumptions: difference to be detected: one scale point, alpha-level: 0.05, power: 80%, and standard deviation: two scale points). For age group II, a sample size of 32 patients was chosen. In order to recruit 160 evaluable patients, about 222 subjects were planned to be screened, due to an assumed screening failure rate of 15% and a drop-out rate of 15%.

### 2.4. Statistical Analyses

The statistical analysis was conducted in compliance with the ICH E9 Note for Guidance on Statistical Principles for Clinical Trials.

The efficacy variables were analysed by using the Mann-Whitney *U* test (differences between groups) and the Wilcoxon test (differences within groups, changes over time), whereby the null hypothesis of no difference between both groups was tested. To evaluate the robustness of results, additional analyses were applied (ANCOVA with baseline values as covariable and sensitivity analyses with several variants of assessments, e.g., using parents' instead of children's assessments). All analyses were performed using Statistical Analysis System software, version 9.2 or higher (SAS Institute Inc., Cary, NC, USA).

## 3. Results

**Figure 2:**
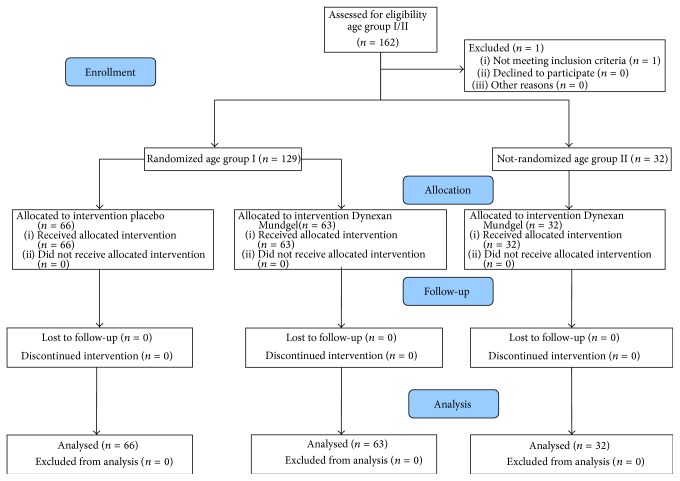
Disposition of study subjects.

Study data were collected between May 21st, 2012, and June 14th, 2014, from 129 patients (age group I) and additional 32 patients (age group II). Within age group I, 63 patients were treated with 2% lidocaine gel (verum) and 66 patients with placebo. No patient dropped out of the study (Figure 2).

### 3.1. Baseline Patient Characteristics

In age group I, 54 male (verum *N* = 30, placebo *N* = 24) and 75 female subjects (verum *N* = 33, placebo *N* = 42) were included. In age group II, both genders were equally distributed (16 male and 16 female patients). The average age in group I was 6.4 ± 1.4 years, ranging from 4.0 to 8.9 years. In age group II, the mean average age was 1.8 ± 0.9 years, ranging from 6 months to 4.0 years. Except for one Black and one Latvian-Korean patient, all other patients were Caucasians.

In age group I aphthous ulcer (36.0%) and in age group II teething (63.6%) were the main cause of painful sites (Table 1).

The mean average size of the painful sites was 19.9 ± 15.7 mm^2^, ranging from 5 mm^2^ to 150 mm^2^. Gingiva and/or oral mucosa were affected in the majority of cases (age group I: 92.7%, age group II: 81.6%), whereas lips or tongue were involved in the remaining cases. The parents in age group I often reported grouching (20.2%), changes in behaviour (24.1%), and poor feeding (26.0%), whereas in age group II weeping (14.2%), crying (13.5%), grouching (18.4%), and sleepiness (18.4%) were often reported.

### 3.2. Pain Assessment

The self-rating by the children of age group I could be used in 107 cases (verum *N* = 53, placebo *N* = 54) at T1 (prior to administration) and in 109 cases (verum *N* = 57, placebo *N* = 52) at T2 (10 ± 5 min after application). The assessments by parents were used in 22 cases at T1 and in 20 cases at T2, because of children's lack of ability to rate their pain properly or showing signs of unwillingness or lack of concentration.

For the secondary efficacy parameter (change of pain rating from T1 to T3 (30 ± 10 min after application)), the self-rating by the children was analysed in 108 patients (verum *N* = 56, placebo *N* = 52) at T3. The assessments by parents were used for 21 patients at T3.

### 3.3. Efficacy Results

On average (median) pain has been reduced by 2.0/5 (verum) versus 1.2/5 (placebo) (Table 3). Nonparametric analysis (Mann-Whitney  *U* test) of treatment related difference in pain assessment yielded a statistical significance (*p* < 0.001) of the observed effect in favour of the 2% lidocaine gel (verum). A similar result (*p* < 0.002) was achieved also for the measurement of pain reduction from T1 to T3. These results were reassured also if the assessment of the same person (either child or parent) was evaluated at both endpoints.

The children's satisfaction as assessed by parents on a 5-point verbal rating scale one hour after administration tends to yield a higher proportion of verum patients showing satisfaction, although observed differences compared to placebo arm did not reach a statistical significance at *α* = 0.05 level (*χ* ^2^-Test including age group II subjects: *p* value = 0.060; *χ* ^2^-Test age group I only: *p* value = 0.259).

Age group II (aged <4 years) patients were all treated with lidocaine 2% gel (verum). In order to assess the efficacy, the individual shift in pain rating had been evaluated. There were no patients with a worsening of pain, 4 patients (12.5%) without a change, 13 patients (40.6%) with an improvement of one, 7 patients (21.9%) of two, 5 patients (15.6%) of three, and 3 patients (9.4%) of four categories in pain rating on T2 compared to T1 (Table 3). This improvement in pain rating was even more pronounced at T3, with 16 patients (50%) indicating “No Hurt,” 13 patients (40.6%) indicating “Hurts Little Bit,” and 3 patients (9.4%) indicating “Hurts Little More” (Table 2). The observed ratings consequently showed statistically significant reduction of pain (Wilcoxon signed rank test results; T2: *p* value < 0.0001 and T3: *p* value < 0.0001).

### 3.4. Safety Evaluation

Seven out of 161 patients (4.4%) reported in total 7 adverse events (worsening of hand-mouth-foot disease, stomach ache, minimal lesion of the upper lip after a fall, bronchitis, single loose bowels, stabbing pain mouth due to aggravating hand-mouth-foot disease, and laceration of the gingiva due to a fall). Two adverse events were registered in each treatment arm in age group I and three adverse events in age group II after verum application (no placebo patients treated). None of these adverse events were assessed as related to study drug.

For most patients (post study examination: 97.5%; one day after the study day: 98.1%) the local tolerability has been rated as “very good.”

## 4. Discussion

In general, clinical studies with younger patients are more difficult to conduct not only because of the regulatory situation, but also because of the lower motivation of the children and their parents to participate and less ability of the young patients to understand the study principles [2, 5]. For minor to moderate and usually short-lasting pain situations, the psychological strain is weak, which affects the willingness to participate in a placebo-controlled clinical study, because the benefit seems not to be substantially relevant. Pain perception in children as a primary efficacy criterion is ambitious and the choice of a suitable method is important [6–8].

Most children aged 4-5 years or older can provide meaningful self-reports of pain intensity, depending on training and provision with age-appropriate tools. Children younger than 4-5 years have a tendency to use only the extremes of a scale. Other response sets or biases include giving the same answer to all questions and responding in an upward or downward sequence to successive questions. Pain rating may be affected by their perception of the consequences of the rating, for example, reward for being brave and fear of getting a syringe. Many other cognitive, social, and cultural factors influence pain ratings (e.g., previous experiences, temperament, presence of an audience, and family and community norms for pain expressions) making them idiosyncratic and difficult to interpret [6]. The assessment of efficacy criteria in studies investigating moderate pain relies on the self-assessment of pain. Patient independent criteria, like lab test or ultrasound, cannot be used.

Topically applied locally acting medicinal products are more difficult to investigate than systemically acting products. Self-healing properties and placebo effects are presumably higher. For local skin or mucous membrane defects, the placebo formulations by themselves may have a healing or protecting effect.

To solve these and other obstacles, we performed this study in a clinical trial facility and not in private practices, made sufficient patient enrolment possible with a variety of recruitment arrangements, excluded younger patients from the possibility to receive a placebo, and used an accepted pain rating scale for children, the Wong-Baker FACES Pain Rating Scale [9]. The participating children were trained and asked by the blinded study personnel in a language the child is familiar with. Additionally, to substitute and fortify the child assessment, the parents or legal guardians were asked to indicate on the same scale, how they consider the child's pain.

More than 80% of children in group I (4–8 years) were able to rate pain by themselves whereas for the remaining children self-reporting could not be used. It is interesting that on average children in this age group rated their pain slightly lower than their parents. However, it should be realized that comparing the child's face with the expression on the faces picture is indirect rating and may be sensitive to bias. As expected and anticipated in age group II mostly only the parents' assessment of how they consider the level of the children's pain before and after administration could be evaluated. These children were too young to allow the use of the pain scale.

The medicinal product used in this study is authorized and on the market in Germany and France for many years. In Germany, it can be applied to children without age restriction. In a recently published clinical study in patients between 6 years and 15 years with pain because of clamp placement, oral trauma, or aphthous ulcers, the identical medicinal product (also due to regulatory circumstances called a cream in France) showed significant superiority compared to the respective placebo formulation [10]. In the present study, significant superiority could be measured in children even down to 4 years of age. Additionally descriptive efficacy and safety data in children between 6 months and below 4 years were obtained, including patients with teething pain, one of the major sources of oral pain in children up to 2 years of age.

Lidocaine alone or in combination with another local anaesthetic is a well-known product for local pain reduction [11, 12]. In different clinical studies conducted with children, the efficacy of topically applied lidocaine could be demonstrated [13–16]. But not in every study could the expected positive outcome be achieved, like in a recent study with children from 6 months up to 8 years suffering from acute infectious ulcerative mouth conditions analysing the effect of a 2% lidocaine viscous solution in comparison to a placebo on the amount of ingested fluid within 60 minutes of administration [17]. At least such an efficacy criterion seems questionable to judge the effectiveness of lidocaine.

Seven out of 161 subjects (4.4%) reported in total 7 adverse events (in age group I 2 adverse events in the verum and the placebo arm each, in age group II 3 adverse events after verum treatment and no patients treated with placebo). None of these adverse events were assessed as study drug related, clearly demonstrating the safety of the product.

## 5. Conclusions

The 2% lidocaine gel, as successfully tested in our clinical trial in comparison to a placebo gel with the same composition without lidocaine, is an adequate tool to reduce or prevent pain on the oral mucosa or gingivae, like bites, aphthous ulcers, mouth blisters, cold sores, gingivitis, hand-foot-mouth disease, dental braces, or teething in younger children.

## Figures and Tables

**Table 1:** Cause of painful sites by age group and treatment arm.

Cause of pain	Treatment arm by age group									
	Age group I						Age group II			
	Verum		Placebo		Total		Verum		Total	
	*N*	%	*N*	%	*N*	%	*N*	%	*N*	%
Bites	8	11.4	2	2.9	10	7.2	2	6.1	2	6.1
Aphthous ulcer	28	40.0	22	31.9	50	36.0	2	6.1	2	6.1
Mouth blisters	6	8.6	10	14.5	16	11.5	1	3.0	1	3.0
Cold sores	0	0.0	0	0.0	0	0.0	0	0.0	0	0.0
Partial gingivitis	11	15.7	9	13.0	20	14.4	0	0.0	0	0.0
Hand-foot-mouth disease	1	1.4	3	4.3	4	2.9	5	15.2	5	15.2
Herpes simplex	0	0.0	0	0.0	0	0.0	1	3.0	1	3.0
Teething	10	14.3	12	17.4	22	15.8	21	63.6	21	63.6
Dental braces	4	5.7	3	4.3	7	5.0	0	0.0	0	0.0
Other	2	2.9	8	11.6	10	7.2	1	3.0	1	3.0
Total	70	100	69	100	139	100	33	100	33	100

**Table 2:** Difference in pain assessment (T2-T1) (age group I [≥4 years]).

Difference in pain assessment (T2-T1)	Verum	Placebo
*N*	63	66
*N* (missing)	0	0
Mean	−2.0	−1.2
Standard deviation	1.1	1.1
Standard error	0.1	0.1
Median	−2	−1
1st quartile (25th percentile)	−3	−2
3rd quartile (75th percentile)	−1	−1
Min	−4	−4
Max	0	2

**Table 3:** Pain rating in age group II: change between T1 (baseline) and T2 (10 ± 5 min after application) or T3 (30 ± 10 min after application).

Pain rating at T1		Pain rating at T2 (10 ± 5 min after application)			
(Total *n* = 32)		Scale 0 (No Hurt)	Scale 1 (Hurts Little Bit)	Scale 2 (Hurts Little More)	Scale 3 (Hurts Even More)
Scale 2	7		4	**3**	
Scale 3	18	3	5	9	**1**
Scale 4 (Hurts Whole Lot)	4	1	2	1	
Scale 5 (Hurts Worst)	3		2	1	

Pain rating at T1		Pain rating at T3 (30 ± 10 min after application)			
(Total *n* = 32)		Scale 0	Scale 1	Scale 2	Scale 3

Scale 2	7	2	5		
Scale 3	18	10	6	2	
Scale 4	4	2	1	1	
Scale 5	3	2	1	—	

Bold values: no change compared to baseline.
